# Spatial Distributions, Sources, Potential Risks of Multi-Trace Metal/Metalloids in Street Dusts from Barbican Downtown Embracing by Xi’an Ancient City Wall (NW, China)

**DOI:** 10.3390/ijerph16162992

**Published:** 2019-08-20

**Authors:** Xiaoping Li, Bin Liu, Yu Zhang, Jiwen Wang, Hameed Ullah, Ming Zhou, Liyuan Peng, Ana He, Xu Zhang, Xiangyang Yan, Tao Yang, Lijun Wang, Hongtao Yu

**Affiliations:** 1 Department of Environmental Science, School of Geography and Tourism, Shaanxi Normal University, Xi’an 710062, China; 2 International Joint Research Centre of Shaanxi Province for Pollutant Exposure and Eco-environmental Health, Xi’an 710062, China; 3 School of Chemistry & Chemical Engineering, Shaanxi Normal University, Xi’an 710062, China; 4 School of Computer, Mathematical and Natural Sciences, Morgan State University, Baltimore, MD 21251, USA

**Keywords:** street dust, trace metal/metalloids, health risk, spatial distribution, traffic-related sources, Xi’an

## Abstract

A total of 116 dust samples in downtown within the city wall were collected, and the spatial occurrence, source and health risk status of 19 trace metal/metalloids bound in street dusts (SDs) were systematically investigated. Geochemical maps, associations, risk models and indices were calculated to define levels of distribution, possible natural or anthropogenic sources, ecological and human health risks. It was found that the wide variations of these 19 trace metals would be observed in spatial maps, which indicated strongly anthropogenic activities inputs. Compared to the calculations of the potential ecological risk index of toxic trace metals, Pb (E_r_^i^ = 20.32) ranked at the level of considerable ecological risk. The non-carcinogenic and carcinogenic risk from most trace metals exposed to children and adults were no significant health risks, except for the non-carcinogenic risk of Cr and As to children, and the carcinogenic risk of Cr to adults. The unacceptable risk locations were observed at traffic conjunctions, which should be given attention. The source apportionment results indicated that the trace metals/metalloids Co, Ga, Nb, As, Ni, and Y, coupled with main elements Al, K, Mg, Ca and Si, would possibly originate from “Soil Re-suspension”, whereas Fe, Cu, Rb, La, Ba, Mn, Ti, Ce and Zr were possibly derived from “Brake Wear”. As regards the Na, no valid assumption was formulated about the presence of this element in brake wear, while Cr, Sr, Zn were possibly associated with “Tire Wear”. Comparatively, V would be suggested as a representative source of fuel consumption, and Pb could possibly belong to “Traffic Pigment”. It was noted that the barbican city, surrounded by the Xi’an Ancient City Wall at 12 m high, would trap the trace metal emissions, and consequently increase the health risk for local residents.

## 1. Introduction

Street dusts (SDs) are the distribution of solid particulates in different locations of the street. With rapid industrialization and urbanization and the growth of population, the amount of dust and toxic pollutions in this dust are increasing [1]. SDs receive a large quantity of trace metals/metalloid inputs from various sources, including industrial emissions such as power plants, coal combustion, the metallurgical industry, auto repair shops [2], urban transportation [3], mining and smelting operations, municipal waste disposal, vehicle exhaust, brake wear and consumer products like lead paint [4,5,6,7]. So, SDs are a more suitable indicator of urban environmental quality than the conventional monitoring of the single air, water and soil, due to the fact that they reflect pollutions from multiple sources [8,9].

It would be proven that SDs associated with trace metal/metalloids can have a negative impact on human health through the hand-mouth pathway, inhalation and dermal contact [1,10,11,12,13]. In the local environment, SDs not only affect the living environment of the habitation, but also cause secondary pollution to the soil, plants and water after settlement [14]. Skin contact and hand-mouth contamination could be the main exposure pathway for humans, for example, children’s unintentional uptake when they play in the playgrounds and city streets [15]. It is reported that lead (Pb) poisoning in children is significantly associated with exposure to SDs associated with the Pb contaminant [16,17].

In recent years, the distribution pattern and risk of trace metal/metalloids in SDs on human health has attracted widespread attention [4,18,19,20,21,22]. However, it was found that the research areas with highly elevated trace metal/metalloids concentrations were generally located in industrial areas, residential zones, roadside and crowded commercial districts. The trace metal/metalloids bound in SDs from downtown in bustling metropolis, their sources were mainly associated with traffic emissions possibly, such as vehicle exhaust particles [23,24], tire wear particles [24,25], street surface particulates, brake-lining emitted particles [24,25], migrated emissions form industrial (power plants, coal combustion, metallurgical industry, auto repair shops and chemical plants, etc.) [23,26], weathering of building and pavement surfaces, and other activities such as city waste incineration. 

Xi’an is a famous city in the world for its long history and splendid culture. It is the capital of the Shaanxi province, and one of the three international cities in northwestern China. Xi’an has experienced a rapid development of its population and economy in recent decades, and the significant release of contaminants, such as Cd, Cu, Pb, and Zn [20,27] into the urban environment placed great pressure on the Xi’an local environment. Despite the fact that studies of trace metal/metalloids contamination in SDs have been conducted in Xi’an before [20,28,29], the researches focused only on a limited trace metal with high toxicity (such as Pb, Zn, Cd, Cr, As). In addition, little attention has been given to downtown areas, which have experienced the long historic accumulation of trace metal/metalloids, and this with high density populations and strongly anthropogenic inputs. The well built-up city wall has protected and stopped the outside pollutants entering the inner city. This special structure of the city wall kept the downtown like a barbican, isolated from other new developed districts in Xi’an city. Meanwhile, in order to well preserve the Xi’an ancient city wall, the industries have been moved out for the past ten or more decades, and all of the public transportation including taxis would be use clean energy. So, the traffic emissions would be the main source of metals/metalloids and would have a strong influence on their distributions in SDs within the ancient city wall. The mixture of hydrocarbons and fine particulate material, including of metals/metalloids were the main emissions from vehicle processes (e.g., engine, tires, brakes), and the climatic conditions could also play an important role in the way of spray and atmospheric deposition of the mixture emissions in the barbican environment. The mixture emissions in the local atmosphere would possibly commence chemical reactions and produce the formation of secondary aerosol particles and haze, and cause adverse health risks to local residents. 

Therefore, the present study was systematically conducted to: (1) Determine the spatial occurrences of multi-trace metal/metalloids accumulated (including important 19 trace metal/metalloids) in SDs within the Xi’an ancient city wall; and (2) to discriminate the potential anthropogenic sources, and (3) to assess the health risk associated with toxic metals in dust originated from traffic sources. The results of this research would provide an important insight into haze control from multi-trace metal/metalloids emissions in the urban environment, and was conducive to the scientific society for human risk management, the local enterprises and the policy makers of the municipality.

## 2. Materials and Methods

### 2.1. Study Area

Xi’an is situated at the central part of the Guanzhong Plain (107.40°–109.49° E, and 33.42°–34.45° N). It belongs to the semi-humid monsoon climate, and has moderate rainfall and four seasons. The annual average temperature is between 13–13.7 °C, and the annual average precipitation between 522.4–719.5 mm. Nowadays, Xi’an has 12 million permanent residents and nearly 3 million cars [30], the highest density of population and heavy traffic activity areas are in the downtown surrounded by the ancient city wall.

The Xi’an city wall, built up in 1374–1378, is the greatest and best preserved ancient city wall in China, is more than six hundreds years old and has experienced being the capital of 13 dynasties. The ancient city wall is 12 m high and 13.7 km long (a regular rectangle), and is surrounded 11.3 square kilometers. The downtown, like a barbican surrounded by the city wall has been the center of the political, economic, cultural, and of trade, from ancient times to nowadays. So the SDs downtown within the ancient city wall are the satisfied “fingerprints” to record and reflect the environment changes and anthropologic activities inputs.

### 2.2. Sampling and Analytical Procedure

**Figure 1:**
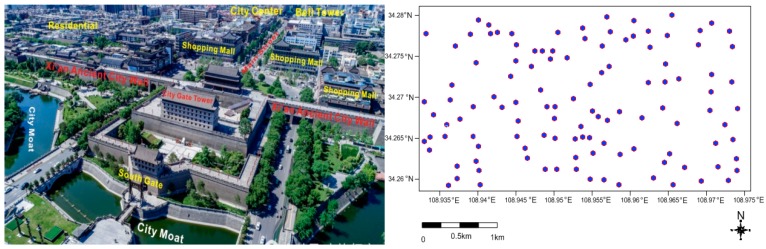
The distribution map of dust sample sites. (City picture source from Qi e hao Da lv xing jia).

SDs samples were collected in the dry season of November 2014 and were collected with plastic brushes and new small steel shovels (not to have any trace metal contamination). A total of 116 dust locations were plotted into Figure 1. Each sample was about 300 g, sealed in polyethylene plastic bags, and marked and transported to the Lab of the International Joint Research Centre of Shaanxi Province for Pollutant Exposure and Eco-environmental Health. Before element analysis, the samples were placed in a dry place for a few days, and then a 2 mm sieve was used to remove greater particles of impurities such as stone. After agate mortar grinding, then, weighing 4 g of the grinding sample into the 32 mm mold to squeeze a tablet with boric acid edge under 30 ton pressure, and preparing for chemical analysis. The element composites were measured by X-ray fluorescence spectrometer (XRF, PANalytical PW-2403 apparatus, PANalytical B.V., Almelo, Holland) [4,31,32]. 

The quality assurance and quality control (QA/QC), the duplicates, method blanks and standard reference materials, were analyzed. A series of soil, sediment, and rock standards, including the GSS-, GRS- and GSD-series geochemical reference materials (Institute of Geophysical and Geochemical Prospecting, Langfang, China), NIST-2709, NIST-2710, NIST-2711 (National Institute of Standards and Technology, USA), and soil standards SO-1, SO-2, SO-3 and SO-4 (Canadian Certified Reference Materials Project), were used to calibrate the instrument. The accuracy and precision of SD analysis was assessed by using standard and duplicate samples with routine ranges from 3% to 5% Relative Standard Deviation (RSD).

### 2.3. Experiment and Analysis Methods

#### 2.3.1. Potential Ecological Risk Assessment Methods

The potential ecological risk was evaluated with the models introduced by Hakanson [33] (1980). The calculating formulas were listed below:

(1) $$C_{f}^{i}=C_{s}^{i}/C_{n}^{i}$$

(2) $$C_{d}=\sum_{i=1}^{n}C_{f}^{i}$$

(3) $$E_{r}^{i}=T_{r}^{i}\times C_{f}^{i}$$

(4) $$RI=\sum_{i=1}^{n}E_{r}^{i}=\sum_{i=1}^{n}T_{r}^{i}\times C_{f}^{i}=\sum_{i=1}^{n}T_{r}^{i}\times \left(C_{s}^{i}/C_{n}^{i}\right)$$

where $C_{f}^{i}$ was the single pollution coefficient of element *i*, $C_{s}^{i}$ was the concentration value of the element *i*, $C_{n}^{i}$ was the geochemical background reference value in the road dust, and in this paper, the background values of metals in Xi’an were adopted from the research data from Cheng et al. [34], *C_d_* was the comprehensive pollution coefficient of metals, $E_{r}^{i}$ was the single potential ecological risk index of element *i*, $T_{r}^{i}$ was the toxicity coefficient of the element *i*, and it reflected the toxicity level of element *i* and the sensitivity of organisms to their pollution, and *RI* was potential ecological risk assessment index of various PTEs. The classification range of *Cd*, $E_{r}^{i}$ and *RI* were related to the type and quantity of the pollutants. The classification of potential ecological risk is listed in Table S1 (in the Supplementary Materials).

#### 2.3.2. Population Health Risk Assessment Methods

Human exposure risk assessment in this study was recommended by the United States Environmental Protection Agency (EPA) [35,36,37,38]. The EPA risk models were the most widely used to assessment the metals’ potential risk [4,32,39]. The long-term average daily exposure to heavy metal intake through the hand-mouth intake route was as follows:

(5) $$ADDing=\frac{C\times ingR\times CF\times EF\times ED}{BW\times AT}$$

The long-term average daily exposure to metals intake through breathing was the following:

(6) $$ADDinh=\frac{C\times inhR\times EF\times ED}{PEF\times BW\times AT}$$

The long-term average daily exposure to metals intake by adsorption through the epidermis was:

(7) $$ADDderm=\frac{C\times SA\times CF\times SL\times ABS\times EF\times ED}{BW\times AT}$$

The average daily exposure of carcinogenic metals was:

(8) $$\begin{array}{l} LADDinh=C\times \frac{EF}{PEF\times AT}\left(\frac{inhR_{child}\times ED_{child}}{BW_{child}}+\frac{inhR_{adult}\times ED_{adult}}{BW_{adult}}\right)\\ C_{95\%UCL}=\exp(\bar{X}+0.5\times s^{2}+s\times H/\sqrt{n-1})\\ C_{95\%UCL}=\bar{X}+t_{a,n-1}\frac{S}{\sqrt{n}} \end{array}$$

(9) $$\begin{array}{l} HQ_{\mathrm{ij}}=\frac{ADD_{ij}}{RfD_{ij}}\\ HI_{ij}=\sum HQ_{ij}\\ RISK_{ij}=LADD_{ij}\times SF_{ij} \end{array}$$

To overcome the uncertainty associated with any estimate of the exposure concentration, an estimate of “reasonable maximum exposure” was usually calculated as the 95% upper confidence limit (95% UCL) of the arithmetic mean. The USEPA’s Superfund program routinely used this procedure to evaluate exposure at hazardous sites; that was, the 95% UCL was commonly used as a public health protective estimate of the true annual average. The significant of all paraments were listed in Table S2. Hazard Quotient (HQ) represented a non-carcinogenic risk, Hazard Index (HI) was the risk index of a variety of exposure ways of a certain pollutant. If the value of HI < 1, it was believed there was no significant risk of non-carcinogenic effects. If HI > 1, then there was a chance that non-carcinogenic effects might occur. With a probability which tended to increase as the value of HI increased. For carcinogens, the lifetime average daily dose (LADD) used in assessing cancer risk had been calculated as a weighted average for each exposure route, as shown in Equation (8). Cancer risks were estimated as the incremental probability of an individual developing cancer over a lifetime since exposure to a potential carcinogen. For its estimation, the LADD was multiplied by the corresponding potency factor to produce a level of cancer risk. Carcinogenic risk was the probability of an individual developing any type of cancer from lifetime exposure to carcinogenic hazards. The acceptable or tolerable risk for regulatory purposes was in the range of 1 × 10^−6^–1 × 10^−4^. R_f_D as a non-carcinogenic reference dose of pollutants, SF was a carcinogenic slope factor. The values of R_f_D and SF were shown in Table S3 (in Supplementary Materials).

#### 2.3.3. Statistical Analysis and Geochemical Mapping

Statistical analysis was carried out using SPSS software (version 21, SPSS Inc., Chicago, IL, USA). The principal components analysis (PCA) and factor analysis (FA) were applied to elucidate about the relationships among metals, and to aid in source identification. Varimax normalized rotation was applied in the PCA analysis. In addition, the correlation coefficients of the metals were calculated to determine the relationships among the elements. Spatial distributions of metal concentrations and risk were depicted through Surfer 11 (Golden Software, Inc., USA).

## 3. Results

### 3.1. The Concentrations of Multi-Major and Multi-Trace Metal/Metalloids in SDs

The descriptive statistics of 7 major metals (metal oxide) and 19 trace metal/metalloids concentrations of SDs within the ancient city wall were presented in Table 1. The mean contents of CaO, Fe_2_O_3_, Na_2_O in the SD of the study area were higher than the background value of the soil in Xi’an, while the contents of Al_2_O_3_, K_2_O, MgO, SiO_2_ were lower than the background value of the soil in Xi’an. The average value of Ba, Ce, Cr, Cu, Pb, and Zn in the SD exceeds the soil background value in Xi’an, and the accumulation of Pb, Zn and Cr was 4.06, 3.89 and 2.34 times more than background values, respectively, which indicated that the contents of these elements were greatly influenced by human activities. Although the average values of others were lower than the background values of Xi’an, most of the metal concentrations nearly approached the threshold values, indicating that continuous anthropolgic activities had a heavy burden on the environment. The observation was demonstrated by the greater variation among Standard Deviation (S.D.) and Coefficient of Variation (C.V.) in Table 1. Comparing SD data from other cities in the world (Table S4 in Supplementary Materials), the mean concentrations of As, Ba, Ce, Cr, La, Nb and V in SDs within the ancient city wall were higher than in other cities (except Cr in Xining), which indicated that the higher contributions of those elements to SDs was possibly associated with human inputs. However, the concentrations of most metals in other cities except Xining did not report. To anthropologic metals, the mean concentrations of Cu, Ni, Pb, Zn in the present study were lower than for all cities, except Cu in Xining, Pb in Xining and Tokyo, Zn in Xining and Banja Luka, Serbia. The big differences of metal concentrations in SDs between cities indicated that they would possibly be influenced by different sources. According to the released data of element concentration in Table S4 (in the Supplementary Materials), the mean concentrations of other major and trace elements in SDs in Xi’an seemed between or above those in other cities.

### 3.2. Spatial Distribution of Multi-Trace Metal/Metalloids in SDs

**Figure 2:**
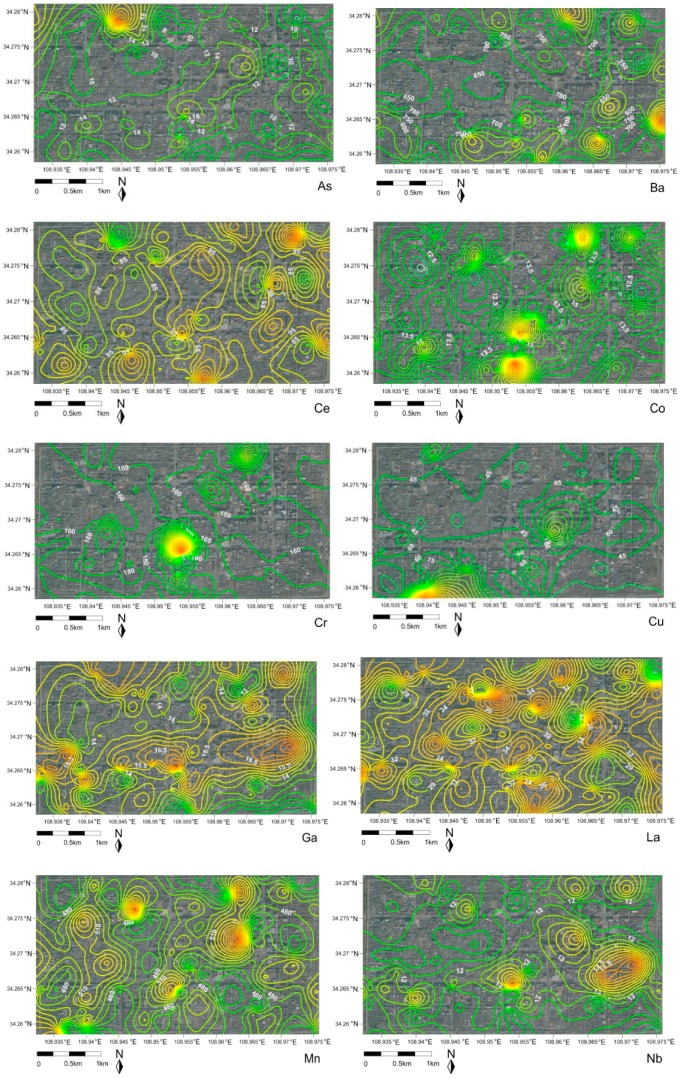

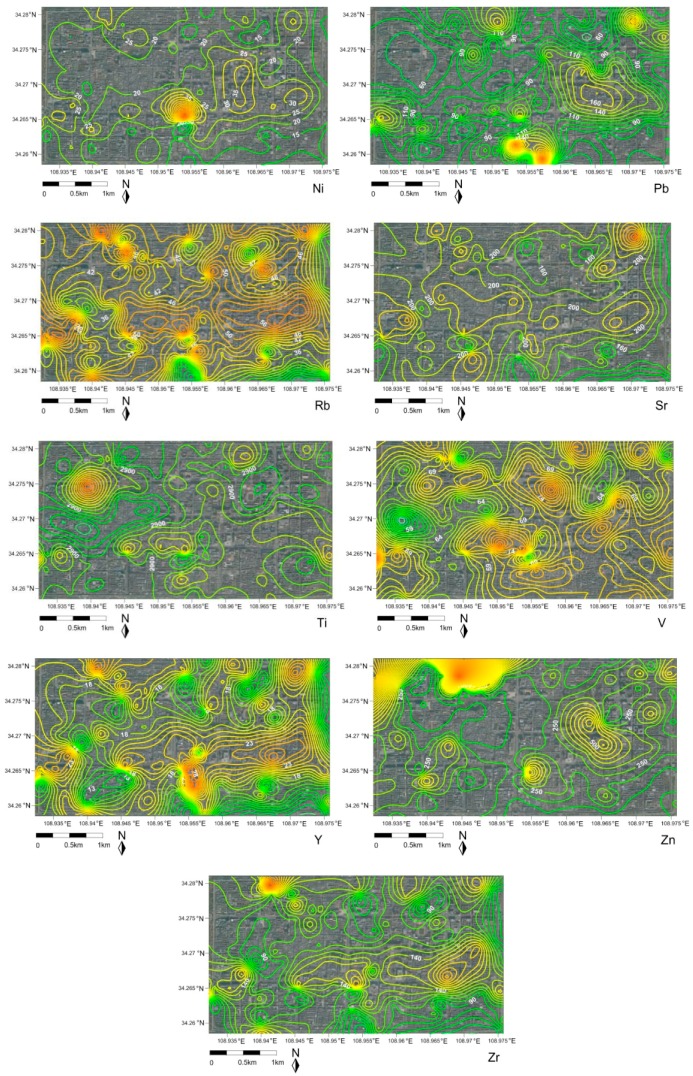
The contour geo-map of trace metal/metalloids in street dust.

The contour map of trace metal/metalloids in street dust was plotted in Figure 2 by Kriging interpolation in Surfer 11.0 (Golden Software, Golden, CO, USA). The Figure 2 showed that the scattered light hotspots were observed in spatial maps, obviously for As, Ba, Co, Cr, Cu, Mn, Ni, Ce, Pb and Zn, indicating the point source pollution. However, nearly homogenous distribution patterns of Ga, La, Nb, Rb, Sr, V, Y and Zr seemed to be another source potentially. It was noted that the high concentration location of Cu was found in the vicinity of the Hanguang Gate and in the Provincial Government, which would possibly be associated with heavy transportation of cars. Whereas, Ni was rich in the vicinity of the Bell tower and Zn was of greater value in Xi’an Railway Station at Anding Gate, indicating of traffic and other source contributions. The geochemical maps of trace metal/metalloids showed the spatial variability in varying degrees.

### 3.3. Potential Ecological Risk Assessment of Multi-Trace Metal/Metalloids in SDs

**Figure 3:**
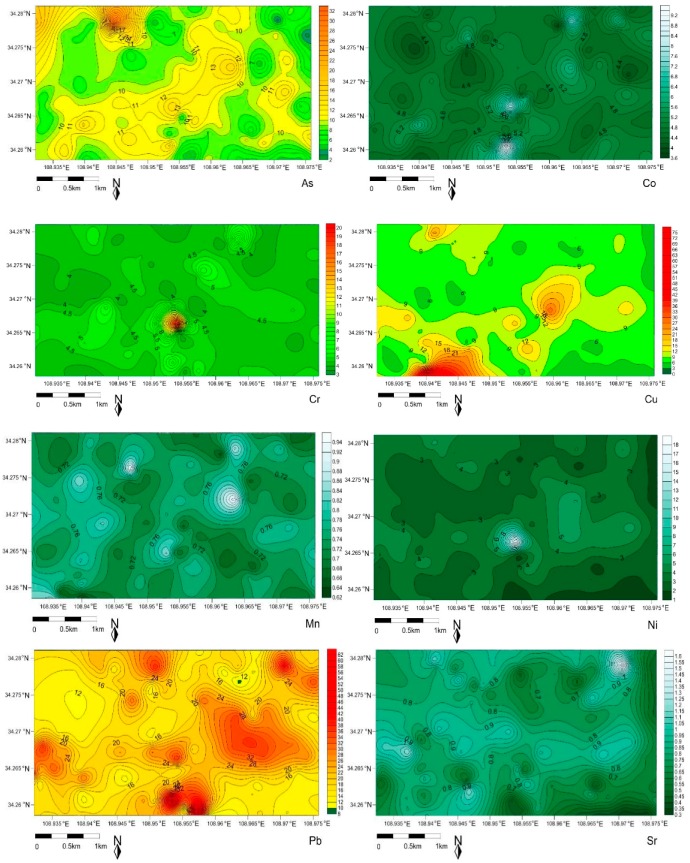

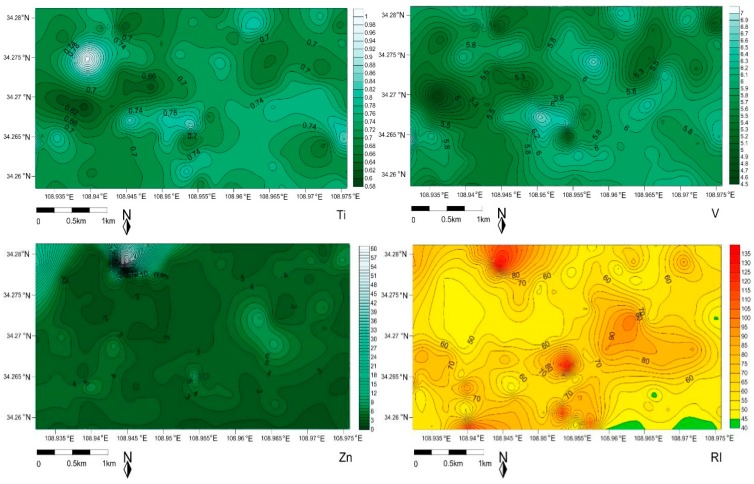
The spatial distribution of potential ecological risk evaluated by the index.

The calculated potential ecological risk index according to the equation (1–4) was listed to the Table S5 and plotted in Figure 3. The results showed that the average value of the single pollution factor of Pb, Zn was more than 3 and less than 6, which belonged to the degree of moderate pollution. Whereas, the indices of trace metals Cr, Cu, V were more than 1 and less than 3, showing this to be at the low pollution level. Comparatively, the values of As, Co, Mn, Ni, Sr, Ti were less than 1 and this indicated they were at the degree of slight pollution. The comprehensive pollution coefficient (C_d_) of 11 kinds of trace metals was 19.9, showing the moderate pollution degree. The average value of the single potential ecological risk index E_r_^i^ of these 11 metals decreased as the order of Pb > As > Cu > V > Co > Cr > Zn > Ni > Sr > Mn > Ti, which indicated Pb (20.32) was at a considerable ecological risk level compared to others. Although the others were at the low risk, the risk value of Cu (9.43) and As (9.78) was near 10, which would be paid more attention, and would need to be continuously monitored. The total potential ecological risk assessment index (RI) of all trace metals was 64.26, belonging to the moderate risk. 

## 4. Discussion

### 4.1. Discrimination the Sources of Multi-Trace Metal/Metalloids in SDs

Principal component analysis (PCA) is the most common multivariate statistical method used in environmental studies, and is employed to extract a small number of latent factors for analyzing relationships among the observed variables [40], and was used to identify the pollution sources, which were generally responsible for the spatial distribution patterns of the metals. Correlation analyses (CA) provided an effective way to reveal the relationships between multiple variables, and thus they were helpful for the understanding of the influencing factors, as well as for the sources of these chemical components [41]. The lognormal distribution was once widely recognized, and was even regarded as a “fundamental law of geochemistry” [42]. 

**Figure 4:**
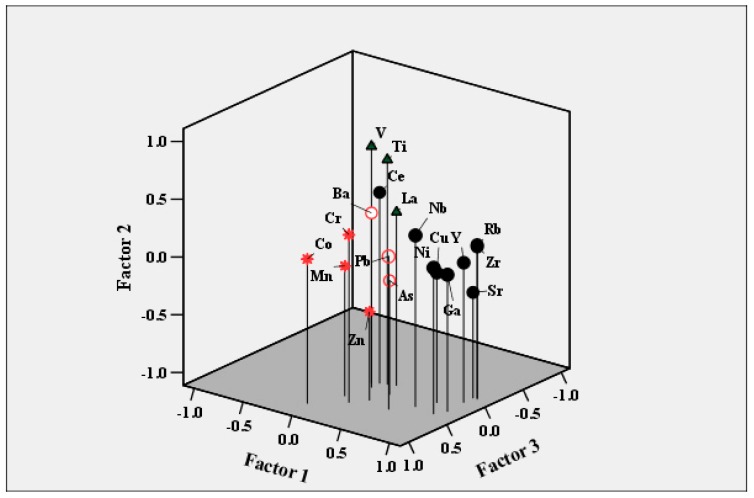
Loadings of the rotated eigenvectors obtained in a principal component analysis (PCA, Varimax rotation method) of the trace metals in SD in Xi’an, NW China.

A log-transformation was carried out to treat these metals statistically using multivariate techniques. Results of Pearson’s correlation coefficients and their significance levels (P < 0.05), of correlation analysis and factor analysis, were shown in Tables S6 and S7 and Figure 4. Four factors were calculated with a cumulative explained variance of about 68%. The first loadings vector (PC1) was characterized by the higher coefficient values of Cu, Ga, Nb, Ni, Rb, Sr, Y and Zr, and this accounted for 34.80% of the total variance. A significantly positive correlation at P < 0.01 was found (Table S5) between the trace metal pairs Ga-Cu (0.71), Ga-Nb (0.69), Ga-Ni (0.90), Cu-Ni (0.70), Sr-Ga (0.80), Rb-Ga (0.84), Y-Ga (0.85) and Ga-Zr (0.86). The high correlated coefficients between metals could be potentially similar sources of pollution. The second factor (PC2) accounted 14.63% for the loadings of Ce, La, Ti and V, with a high correlation between V-Ce (0.55) and Ti-V (0.85). Cr, Co, Mn and Zn attributed to a third factor (PC3) with 11.71%; The fourth factor (PC4) was inclusive of As, Ba and Pb with an explained 6.69%. The trace metals/metalloids Cu, Ga, Nb, Ni, Rb, Sr, Y and Zr loaded in the factor 1 with the relatively high variance% and Ce, La, Ti and V in factor 2 would be attributed to the coal combustion and coal fly ash in the previous studies [43,44,45,46,47,48]. However, in this study, most trace metals were observed at the lower mean concentrations than the local soil background, except Cu and Ce slightly higher, suggesting that the source of these metals resulting from coal combustion was limited. Meanwhile, From hierarchical clustering analysis in Figure S1, and a boxplot of content for trace metals in Figure S2, it was obvious that the trace metals/metalloids would be clustered in five groups, one was a cluster of Co, Ga, Nb, As, Ni, Y, the second was Cu, Rb and La, the third was Ce, V, Pb, Zr, the fourth was Cr, Sr and Zn, and the rest was Ba, Mn and Ti. Usually, Cu, Ti and Zr were the main vehicle emissions via brake pads [49,50] and Cu was also used in brakes to control heat transport [51], which particles emitted from brake wear are suspended in the atmosphere, and then tend to deposit onto the ground [52]. Ce, Rb and La were also usually associated with emitted brake wear dust [25]. Cr was an important constituent of metal alloys (e.g., Cr–V, Cr–W). Ba, Mn and Ti were reported as tracers of braking pads wear [53,54]. Indeed, Ba was used as a filler in brake linings to increase the density of brake pads, and also as an extender in paints (BaSO_4_, baryte). Also, Ti was commonly used in white paint in the form of TiO_2_ and in brake pads (especially in metallic-ceramic compounds). Pb, which was an indicator of vehicle-related metal in urban areas [55], was used as the gasoline additive [56], since it was banned in China in 2000s and replaced by methyl-cyclopentadienyl manganese (MMT) as a petrol additive [57]. However, Pb had a long half-life [58] and low leaching rates [59], resulting in its enrichment in the urban environment over a long time. In addition, the presence of Pb in street dust could be linked to yellow road lines, because PbCrO_4_ was frequently used as a pigment in paints [25,60]. Furthermore, the carbonate host minerals of Pb such as cerussite (PbCO_3_) and Pb hydroxyl carbonate had been widely used as the white pigment [61] in road surface marking. Meanwhile, Zn was commonly linked to lubricating oil and derived from automobile tires [62,63,64]. It was noted that As had the negative value (−0.66), but Ba (0.43) and Pb (0.58) had positive values in Factor 4, respectively. In addition As with Ba and Pb were separated from the other elements in hierarchical clustering analysis in Figure S1, which considering As and Ba, while Pb might originate from different sources. V, in association, was usually recognized as a marker of fuel-oil combustion and petrochemical plants emission [65,66]. The major elements Ca, Fe and Na contents were higher than background values in the soil, CaCO_3_ was a common mineral found in atmospheric particulate, road dust and dust particles. It was usually used as a tracer for re-suspended soil dust, handling of construction materials and building projects [67,68], in addition, Ca and Fe were usually found in the compositions of brake materials [69]. Ca, Ce, Fe, Ti and Na were supposed to be the typical geological marker elements [70]. In this study, the mean concentrations of trace metals/metalloids Ba, Ce, Cr, Cu, Pb and Zn exceeded the soil background, indicating the obvious anthropogenic sources. Although it was unclear the reason why Cu belonged to the Factor 1 with Cu, Ga, Nb, Ni, Rb, Sr, Y and Zr, it was characterized by high Pearson correlation values, demonstrating that it was possibly almost exclusively emitted by a single kind of source. 

Since the Xi’an government stopped industrial production, coal and biomass combustion, as well as having taxi automobiles using clean gas for car engines within the ancient city wall at least 10 years ago, emissions from traffic were claimed to be the main source of trace metals/metalloids. Therefore, based on these assumptions we suggested Co, Ga, Nb, As, Ni, Y and Al, K, Mg, Ca and Si could possibly be originated from “soil re-suspension”. Fe, Cu, Rb, La, Ba, Mn, Ti, Ce and Zr were associated with “brake wear”. Although the Zr mean concentration value was not higher than its background, Zr possessed the high positive correlation at P < 0.01 to Cu (0.7), and for this reason, we suggest that a significant but not exclusive source of these elements was brake wear. Indeed for example, Ce was found in brake wear emission [25]. As regards the Na, no valid assumption was formulated about the presence of this element in brake wear, while Cr, Sr, Zn would possibly be controlled by “tire wear”. A possible explanation of this grouping was that the cruise speed increases the tire wear emission, including the soil particles previously embedded on tire treads [25,71]. Trace metal V would be suggested as a representative source of traffic-related of fuel consumption, and Pb would possibly belong to “traffic pigment in lane paints”.

**Figure 5:**
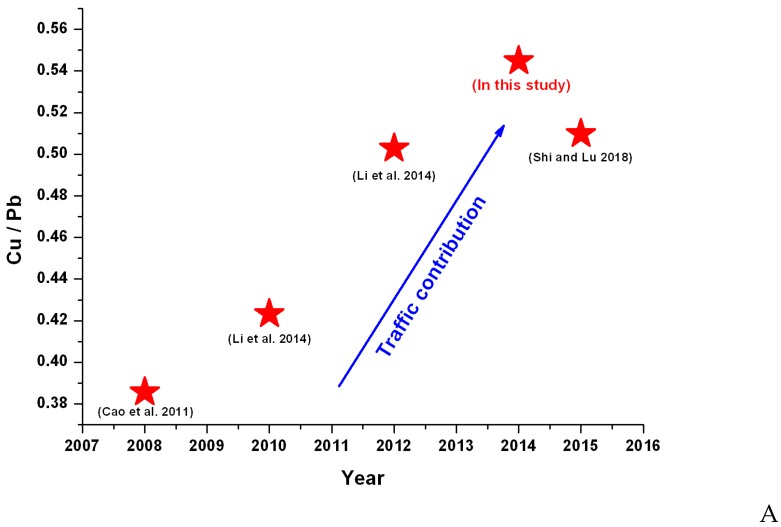

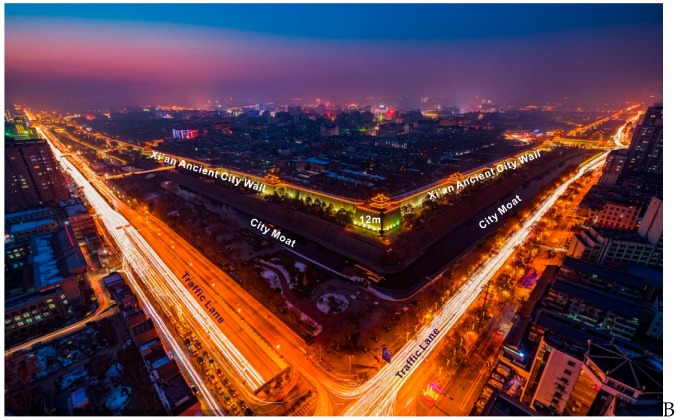
The brake wear contribution (**A**) and the busy transportation map of the Xi’an ancient city wall (**B**). (Picture source from www.vcg.com).

The Cu/Pb concentration ratio was usually employed to identify the contribution of the brake wear source in environmental matrices such as atmospheric particulate and road [66]. Given this approach, the Cu/Pb ratio was plotted in Figure 5A. In the graph, it was found that Cu/Pb in this study was higher than it was in Cao et al. [72], Li et al. [73] and Shi & Lu [74], indicating the more significant portion of traffic contribution within this city wall than out of it. The shape surrounded by the Xi’an Ancient City Wall was a regular rectangle, and the topography of the center looked like a barbican city (Figure 5B).

When thousands of automobiles shuttled every day into the barbican city, surrounded by the 12 m high city wall, traffic emissions, including brake, tire wear and fuel combustion, would be enriched within the city wall, and could not be diffused quickly because of the high city wall preventing this. Meanwhile, the traffic emissions from out of the city wall also could not easily transfer into the inner, which was the reason why the Cu/Pb ratio was significantly higher than others. This observation was also confirmed by the results from Cao et al. [72], indicating the relatively high deposition rate (mg∙m^−2^∙yr^−1^) ratio between Cu and Pb within the city wall site than out of the city. Although the results of the present study provided preliminary conclusions regarding the origin of each metal, further studies were necessary to gain a better understanding of their sources.

### 4.2. Health Risk Assessment of Toxic Trace Metals Potential Emission from Traffic Sources in SDs

**Figure 6:**
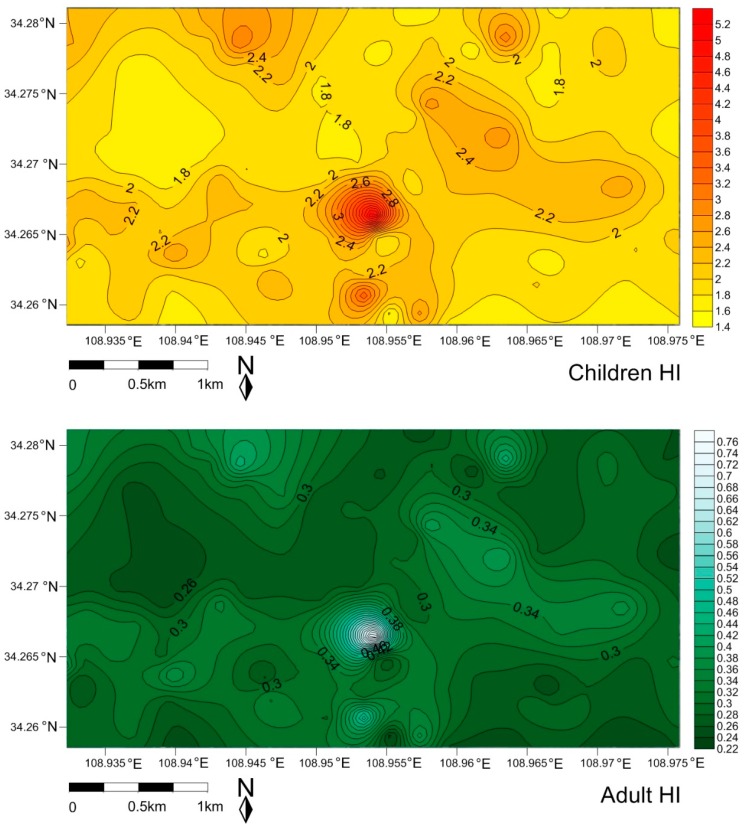
The spatial risk of total non-carcinogenic for children and adult exposure to the traffic-related multi-trace metals/metalloids in street dust.

**Figure 7:**
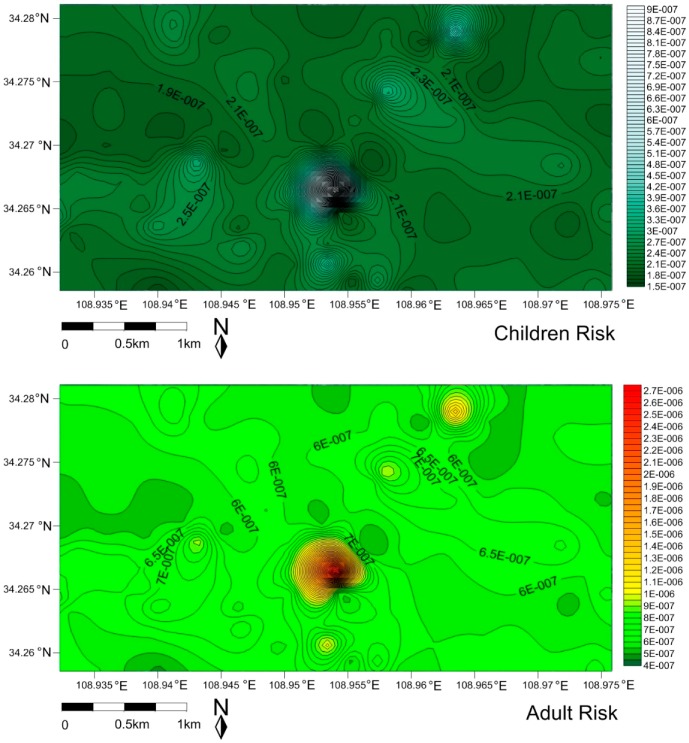
The spatial risk of total carcinogenic for children and adult exposure to the traffic-related multi-trace metals/metalloids in street dust.

The carcinogenic and non-carcinogenic health risks of traffic-related toxic metals/metalloids exposed to children and adults were evaluated by health risk assessment models, and the spatial risk maps were plotted for children (Figure 6 and Figure 7, Figures S3 and S5, Tables S8 and S9 in the Supplementary Materials) and adults (Figure 6 and Figure 7, Figures S4 and S6, Tables S8 and S9 also in the Supplementary Materials). It was found that ingestion of dust particles appeared to be the main exposure pathway for As, Co, Cr, Cu, Ni, Pb, Zn, Mn, Ba, Sr, V to the children and adults group, and the dermal and inhalation pathway followed it, except Co, Ba, Mn for the adults and Co, Mn for the children; which tendency was similar to the observation of the daily exposure doses (Table S8). The daily exposure doses of the children were greater than those of the adults, which was attributed to the fact that that children were more vulnerable to dust exposure because of their playing habits (ingestion of dust through mouth, hand licking, toys and other household objects) [75]. Cr, As, Pb and Ba, Mn, V were the more risky trace metals exposed to children through ingestion. Although the mean value of non-carcinogenic risk (HI) for children and adults were less than 1 and in an acceptable range, Cr and As exposure to children in some hotspots (the HI values were much close to 1, Figure S3) which are located at traffic conjunctions should be paid more attention. The total HI of traffic-related multi-trace metals/metalloids was displayed in the Figure 6. The calculations showed that the non-carcinogenic health risk (beyond the HI threshold) of multi-trace metals/metalloids to children was higher than that of adults, indicating some measures should be taken to reduce the injury to children. 

The carcinogenic risks of As, Co, Cr, Ni and Pb exposure to children and adult groups at different locations were shown in Tables S8 and S9 in the Supplementary Materials. The cancer risk (CR) values for As, Co, Cr, Ni and Pb for adults (1.59 × 10^−8^, 1.17 × 10^−8^, 6.63 × 10^−7^, 1.63 × 10^−9^ and 3.58 × 10^−10^, respectively) were higher than for children (5.40 × 10^−9^, 3.97 × 10^−9^, 2.25 × 10^−7^, 5.55 × 10^−10^, 1.21 × 10^−10^ respectively). However, the mean CR values were between the safe level 1 × 10^−6^ to 1 × 10^−4^, indicating that the carcinogenic risks of As, Co, Cr, Ni, and Pb to children and adults were in the acceptable range. In addition, it was clearly observed that few locations beyond the CR threshold (1 × 10^−6^ to 1 × 10^−4^) of Cr in the case of adults were identified in the carcinogenic risk map with the light color. The calculated values of total carcinogenic risk from traffic-related multi-trace metals/metalloids observation in Figure 7 would possibly be indicated a higher potential carcinogenic risk (beyond the CR threshold) to local adults compared to the children. Certainly, the total carcinogenic risk data were only observations for comparison, and the carcinogenic risk caused from multi-trace metals/metalloids would need to be further identified and discussed. 

Generally, the traffic emissions were believed to be entrained and dispersed by the primary flow vortex and wind tunnel. The city wall looked like a “barbican”, which was used to protect the whole city’s security in ancient times. Nowadays, with rapid urbanization the downtown surrounded by this city wall has become a busy center of shopping, commerce and tourism. Strong velocity and frequency of anthropologic activities, including that of traffic, would happen in there every day. The barbican city surrounded by the City Wall with 12 m of height would be responsible for the “trapping” of emission pollutions, and reduce the dispersal of such pollution. Consequently, it would potentially enhance the exposure risk of toxic emissions to habitations. 

This was an illustrative rather than a generic study, to be followed by more systematic research aimed at generalizing conclusions and understanding processes sufficiently to provide broad design guidance.

## 5. Conclusions

The street dust bound 19 trace metals/metalloids associated with being traffic-related were collected within the Xi’an ancient city wall, and their spatial distribution, traffic-related sources and health risk were systematically evaluated. The wide variations in these 19 trace metals would be observed in spatial distribution maps. Results of the potential ecological risk assessment of trace metal/metalloids in SDs were associated with moderate ecological risk integratedly. Compared to other single metals, Pb (20.32) was at a considerable ecological risk level. Although the non-carcinogenic and carcinogenic risk from trace metals exposed to children and adults were within acceptable ranges, non-carcinogenic risk of Cr and As to children and carcinogenic risk of Cr to adults at traffic conjunctions should not always be ignored. In addition, the unacceptable risk from the traffic-related multi-trace metals/metalloids in SDs exposed to children in non-carcinogenic risk and the total carcinogenic risk for adults were discriminated. It was noteworthy that the trace metals/metalloids Co, Ga, Nb, As, Ni and Y, coupled with the main elements Al, K, Mg, Ca and Si, would possibly be originated from “soil re-suspension”, whereas Fe, Cu, Rb, La, Ba, Mn, Ti, Ce and Zr were possibly derived from “brake wear”. 

As regards the Na, no valid assumption was formulated about the presence of this element in brake wear. While, Cr, Sr, Zn would be possibly controlled by “tire wear”. Comparatively, the trace metal V would be suggested as a representative source of traffic-related of fuel consumption, and Pb would possibly belong to “traffic pigment in lane paints”. The barbican city surrounded by the Xi’an Ancient City Wall would trap the diffusion of trace metals/metalloids from traffic emissions and increase the exposure risk of toxic metals for habitations lived within this city wall, which should garner careful attention.

## Figures and Tables

**Table 1:** Statistical results of chemical elements content in Street dust in Xi’an.

Elements		LLD	Unit	Mean	Minimum	Maximum	S.D.	C.V.	Background Value of Soil in Xi’an [34]
Trace metal/metalloids	As	0.6	mg/kg	11.73	2.60	39.20	4.25	0.36	12.00
	Ba	6.3	mg/kg	748.23	560.70	1366.20	127.75	0.17	542.00
	Ce	14.5	mg/kg	88.59	36.40	141.90	14.65	0.17	68.00
	Co	0.9	mg/kg	13.71	10.50	26.40	2.14	0.16	14.00
	Cr	3.1	mg/kg	175.18	129.40	784.80	66.21	0.38	75.00
	Cu	0.7	mg/kg	50.91	19.70	390.40	38.62	0.76	27.00
	Ga	0.7	mg/kg	14.54	10.80	19.30	1.82	0.13	16.00
	La	7.3	mg/kg	31.92	18.90	42.00	4.01	0.13	40.00
	Mn	1.6	mg/kg	486.42	412.40	622.40	34.68	0.07	660.00
	Nb	0.5	mg/kg	11.90	9.70	18.00	1.38	0.12	15.00
	Ni	0.7	mg/kg	21.04	6.80	122.50	11.79	0.56	32.00
	Pb	1.4	mg/kg	93.45	40.70	289.10	42.76	0.46	23.00
	Rb	0.7	mg/kg	44.19	17.40	65.80	10.24	0.23	106.00
	Sr	0.7	mg/kg	186.54	80.20	380.20	47.64	0.26	242.00
	Ti	8.7	mg/kg	2949.18	2387.00	4162.00	245.52	0.08	4092.00
	V	2.4	mg/kg	69.28	53.20	83.60	4.89	0.07	88.00
	Y	0.8	mg/kg	18.57	9.20	32.60	5.37	0.29	24.00
	Zn	0.8	mg/kg	272.04	102.90	4344.80	416.20	1.53	70.00
	Zr	0.8	mg/kg	120.13	47.40	301.20	38.94	0.32	209.00
Major metals	Al_2_O_3_	0.01	%	8.04	6.87	9.28	0.45	0.06	12.33
	CaO	0.01	%	8.17	3.24	14.28	1.37	0.17	6.33
	Fe_2_O_3_	0.01	%	5.36	4.14	11.2	0.95	0.18	4.75
	K_2_O	0.01	%	1.45	1.09	1.62	0.08	0.06	2.59
	MgO	0.01	%	1.96	1.68	2.72	0.15	0.08	2.26
	Na_2_O	0.01	%	2.52	2.00	3.69	0.26	0.10	1.44
	SiO_2_	0.02	%	37.18	30.19	43.97	2.45	0.07	58.76

## Supplementary Materials

The following are available online at https://www.mdpi.com/1660-4601/16/16/2992/s1, Figure S1: Hierarchical clustering analysis of trace metals in street dusts from Xi’an (Cluster method: Between-groups linkage; Interval: Squared Euclidean distance), Figure S2: Boxplot of the content for trace metals in the SD from Xi’an (number in the figure represents the site of sample), Figure S3: The spatial risk of non-carcinogenic of trace metals/metalloids exposed to children, Figure S4: The spatial risk of non-carcinogenic of trace metals/metalloids exposed to adults, Figure S5: The spatial risk of carcinogenic of trace metals/metalloids exposed to children, Figure S6: The spatial risk of carcinogenic of trace metals/metalloids exposed to adults, Table S1: The potential risk classification, Table S2: Parameters for health risk assessment, Table S3: Reference dose (RfD) and carcinogenic slope factor (SF) for the different exposure routes of toxic metals, Table S4: Comparison of metal concentration in the SD of various cities worldwide (unit in mg∙kg^−1^), Table S5: The potential ecological risk assessment of street dust, Table S6: The correlation analysis of metals in Street dusts, Table S7: Varimax rotated factor matrix, Table S8: Daily exposure levels of children and adults to trace metals in dust under different exposure routes, Table S9: The risk index of children and adults of trace metals in dust under different exposures.
