# Supplementary material for: Spatial Distributions, Sources, Potential Risks of Multi-Trace Metal/Metalloids in Street Dusts from Barbican Downtown Embracing by Xi’an Ancient City Wall (NW, China)
Source: Int J Environ Res Public Health. 2019 Aug 20;16(16):2992. doi: 10.3390/ijerph16162992 (PMC6727082; doi:10.3390/ijerph16162992)
Supplement: Supplementary file 1 [file ijerph-16-02992-s001.zip › ijerph-514983-supplementary/ijerph-514983-figures.pdf]

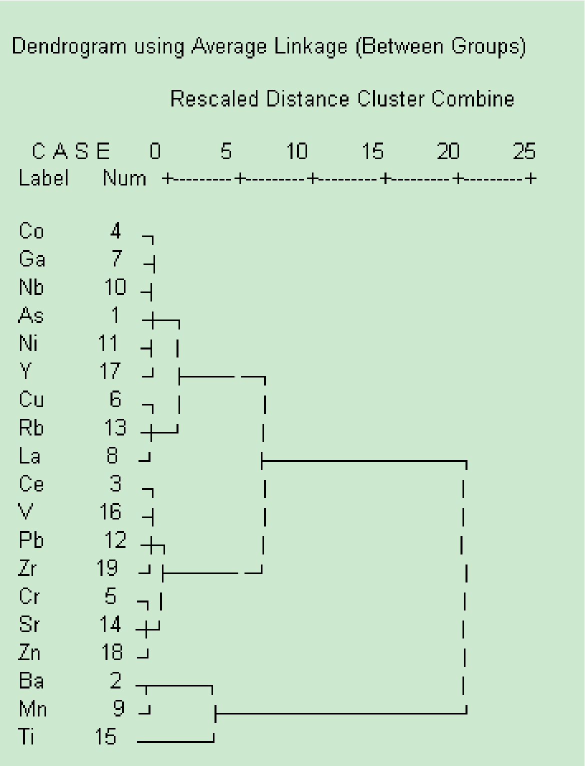

Fig.S1 Hierarchical clustering analysis of trace metals in street dusts from Xi'an

(Cluster method: Between-groups linkage; Interval: Squared Euclidean distance)

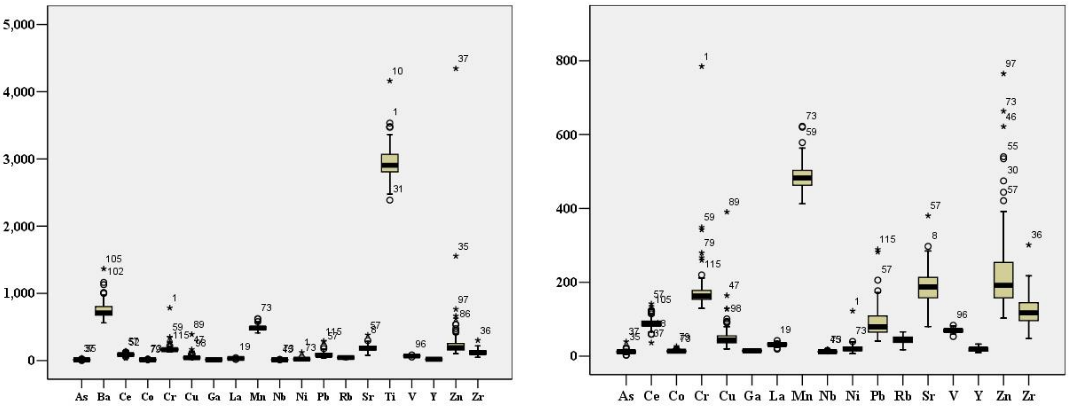

Fig.S2 Boxplot of content for trace metals in the SD from Xi'an

(number in the figure represents the site of sample)

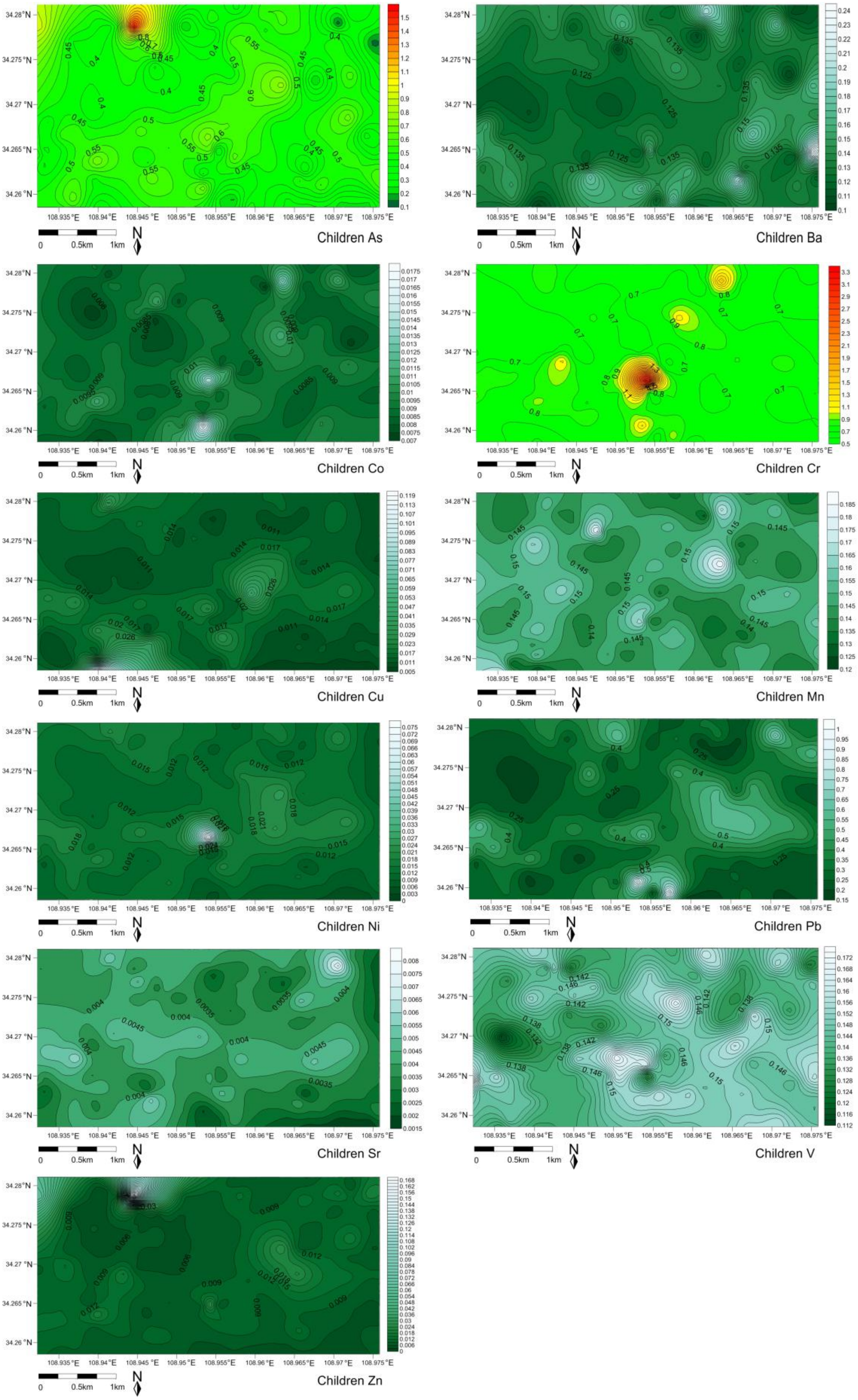

Fig. S3 The spatial risk of non-carcinogenic of trace metals/metalloids exposured to children

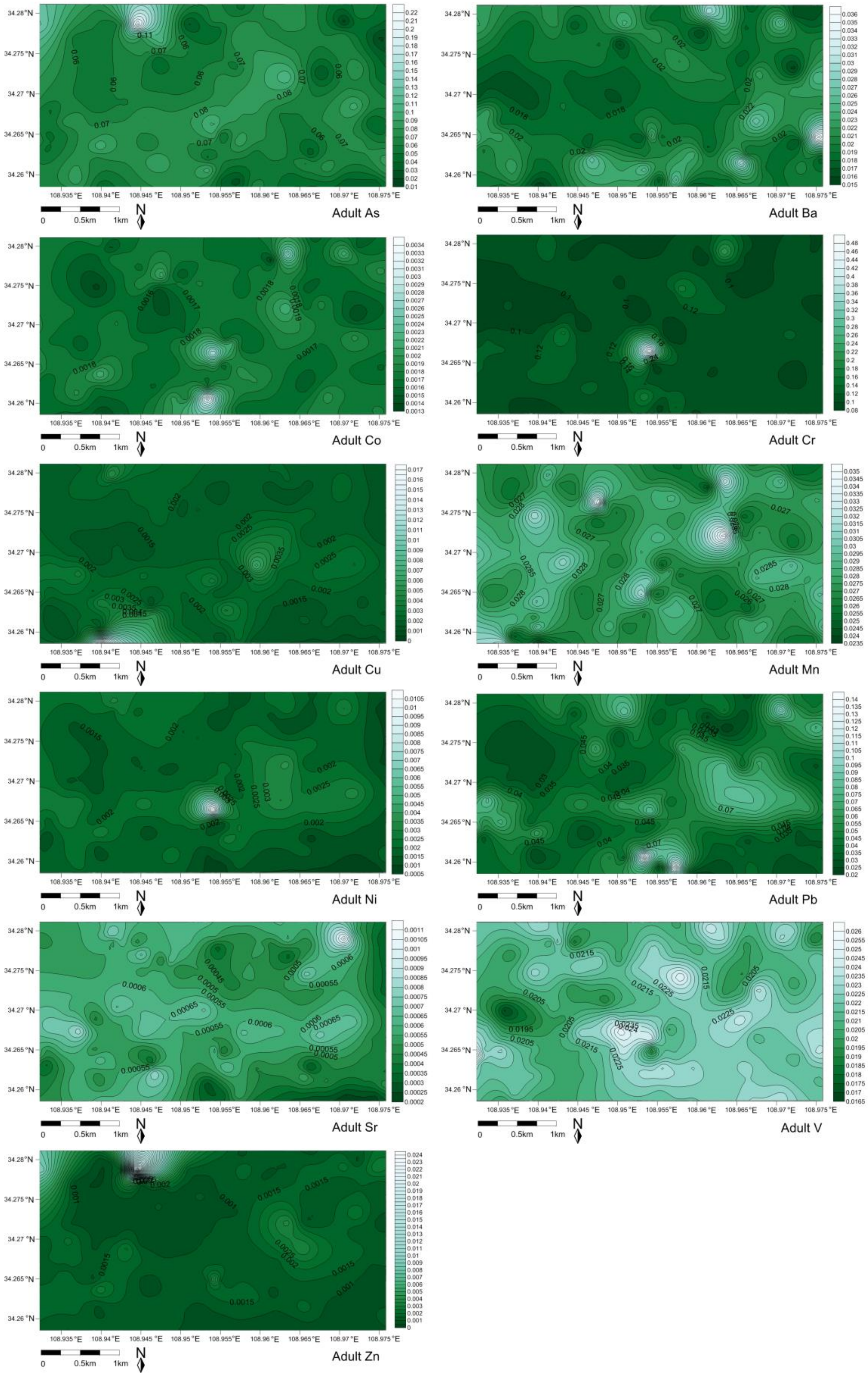

Fig. S4 The spatial risk of non-carcinogenic of trace metals/metalloids exposured to adults

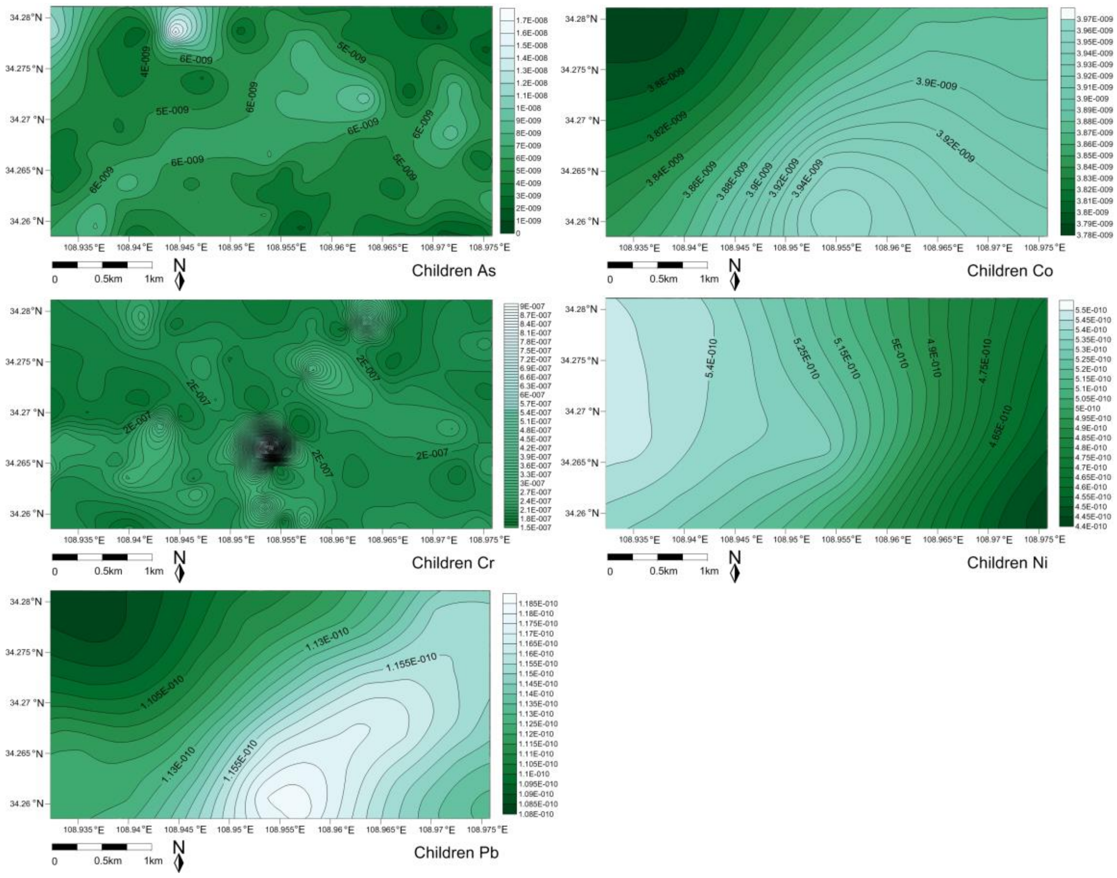

Fig.S5 The spatial risk of carcinogenic of trace metals/metalloids exposured to children

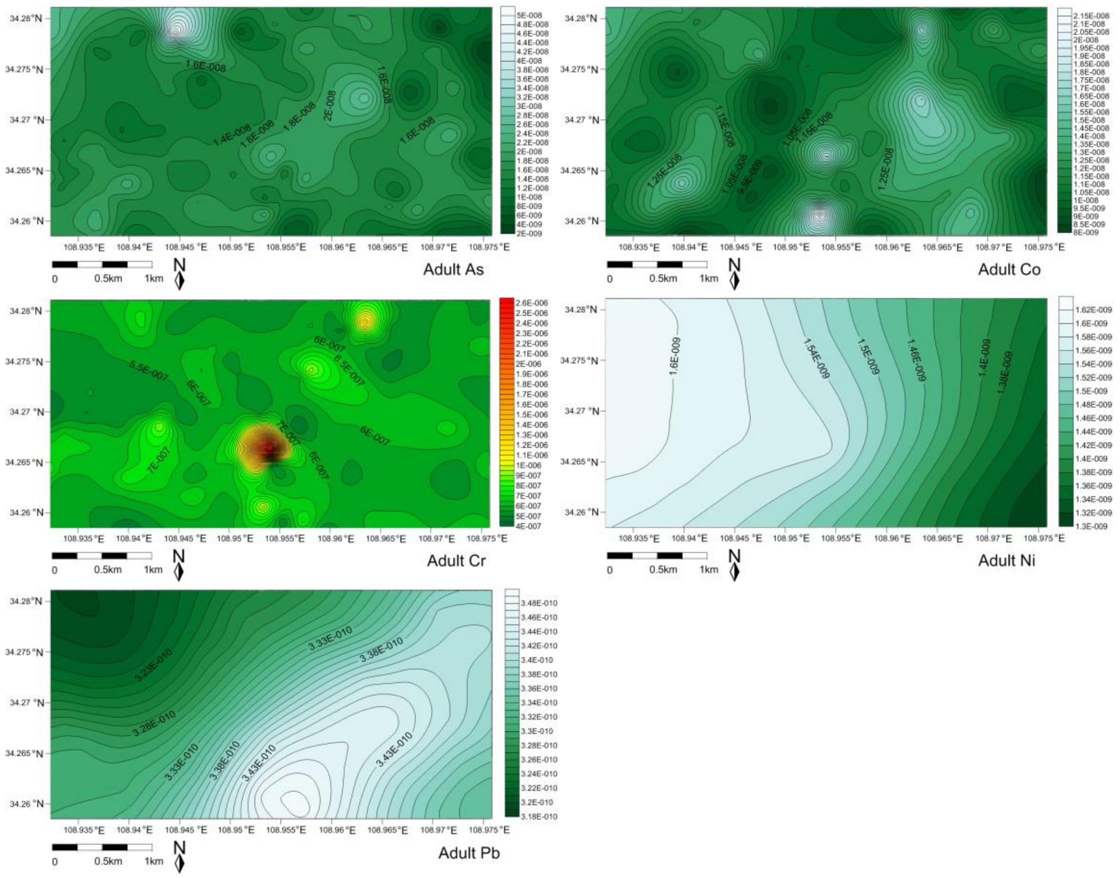

Fig.S6 The spatial risk of carcinogenic of trace metals/metalloids exposured to adults
