# Supplementary material for: Spatial Distributions, Sources, Potential Risks of Multi-Trace Metal/Metalloids in Street Dusts from Barbican Downtown Embracing by Xi’an Ancient City Wall (NW, China)
Source: Int J Environ Res Public Health. 2019 Aug 20;16(16):2992. doi: 10.3390/ijerph16162992 (PMC6727082; doi:10.3390/ijerph16162992)
Supplement: Supplementary file 1 [file ijerph-16-02992-s001.zip › ijerph-514983-supplementary/ijerph-514983-tables.pdf]

Table S1 The potential risk classification

Type	Degree of risk				
	Low risk	Moderate risk	Considerable risk	High risk	Extremely risk
RI	<40	40~80	80~160	160~320	>320
E_r^i	<10	10~20	20~40	40~80	>80

Type	Degree of pollution			
	Slight	Low	Moderate	Considerable
C_f^i	<1	1~3	3~6	>6
C_d	<11	11~22	22~24	>44

Table S2 Parameters for health risk assessment

Parametere	Significant	Unit	Value
ADD_{ing}	Daily exposure amount of metals through ingestion	mg/kg•d^{-1}	
ADD_{inh}	Daily exposure amount of metals through inhalation	mg/kg•d^{-1}	
ADD_{derm}	Daily exposure amount of metals through dermal contact	mg/kg•d^{-1}	
LADD	Lifetime average daily dose	mg/kg•BW•d^{-1}	
HQ	Hazard quotient	dimensionless	
HI	Hazard Index	dimensionless	
RISK	Cancer risk	dimensionless	
95%UCL	95% upper confidence limit		
X	Arithmetic mean of log-transformed data		
s	Standard deviation of log-transformed data		
H	H-statistic		
n	Number of samples		
C	Exposure level of metal in dust	mg/kg	95%UCL
IngR	Intake frequency of hand-mouth	mg/d	200(children)100(adults)
InhR	Breathing rate	m^3/d	7.6(children)20(adults)
CF	Conversion coefficient	m$^{3/}$mg	1×10^{-6}
EF	Exposure frequency	d/a	350
ED	Exposuree time	a	6(children)24(adults)
BW	Average weight	kg	15.9(children)56.8(adults)
AT	Average total exposure time	d	ED×365d(non-carcinogenic)70 ×365(carcinogenic)
PEF	Particulate matter emission factor	m^3/kg	1.36×10^9
SA	Axposure of skin surface area	cm^2	2160(children)4220(adults)
SL	Skin sticky limit	mg/(cm^2.d)	0.2(children)0.07(adults)
ABS	Skin absorptive factor		As is 0.03, the rest is 0.001

Table S3 Reference dose (RfD) and carcinogenic slope factor (SF) for different exposure routes of toxic metals

Metals	As	Co	Cr	Cu	Ni	Pb	Sr	Zn	Ba	Mn	V
RfD_{ing}	3.00E-04	2.00E-02	3.00E-03	4.00E-02	2.00E-02	3.50E-03	3.00E-01	3.00E-01	7.00E-02	4.60E-02	7.00E-03
RfD_{inh}	3.01E-04	5.71E-06	2.86E-05	4.02E-02	2.06E-02	3.52E-03	3.00E-01	3.00E-01	1.43E-04	1.43E-05	7.00E-03
RfD_{derm}	1.23E-04	1.60E-02	5.00E-05	1.20E-02	5.40E-04	5.25E-04	6.00E-02	6.00E-02	4.90E-03	1.84E-03	7.00E-05
SF_{inh}	1.51E+01	9.80E+00	4.20E+01		8.40E-01	4.20E-02					

Table S4 Comparison of metals concentration in the SD of various cities worldwide (unit in mg·kg^{-1})

City	CaO	Fe_2O_3	K_2O	MgO	Na_2O	SiO_2	Al_2O_3	As	Ba	Bi	Ce	Co	Cr	Cu
Xi'an, China [a]	8.17%	5.36%	1.45%	1.96%	2.52%	37.18%	8.04%	11.73	748.23		88.59	13.71	175.18	50.91
Xining, China[b]	6.06%	4.22%	1.17%	2.29%	2.76%	30.9%	5.2%	6.00	344.43	2.86	61.56	24.6	507.77	30.32
Beijing, China[c]	63.9g/kg	27.3g/kg	13.7g/kg	14.9g/kg	11.2g/kg		45.7g/kg		593			11.5	72.1	98.0
Xi'an, China[d]								10.62					167.28	94.98
Delhi, India[e]													148.8	191.7
Buenos Aires, Argentina[f]		10.97 g/kg		1.09 g/kg			12.71 g/kg		346				91	190
Banja Luka, Serbia[g]												63.1	34.6	77.7
Tokyo, Japan[h]													52.3	
Tokyo, Japan[i]	2.25%Ca	1.00%Fe	0.69%K	0.4%Mg	0.83%Na		1.57% Al		208			4.69	67.8	304

[a] This study.

[b] Li, XP, ZhangM, GaoY, ZhangYC, Zhang X, Yan XY, Wang S, Yang R, Liu B, Yu HT, (2018). Urban street dust bound 24 potentially toxic metal/metalloids (PTMs) from Xining valley-city, NW China: Spatial occurrences, sources and health risks, Ecotoxicol. Environ. Safe. 162, 474-487

[C] Tang, Y., Han, G.L., (2017). Characteristics of major elements and heavy metals in atmospheric dust in Beijing, China. J. Geochem. Explor. 176, 114-119.

[d] Yongming, H., Peixuan, D., Junji, C. and Posmentier, E.S., (2006). Multivariate analysis of heavy metal contamination in urban dusts of Xi'an, Central China. Sci. Total Environ., 355(1-3), 176-186.

[e] Suryawanshi, P.V., Rajaram, B.S., Bhanarkar, A.D., Chalapati, Rao, C.V., (2016). Determining heavy metal contamination of road dust in Delhi, India. Atmósfera 29 (3), 221-234.

[f] Fabián, G.F., Darío, R.G., Laura, D., Patricia, P., Ana, F., (2011). Metals associated with airborne particulate matter in road dust and tree bark collected in a megacity (Buenos Aires, Argentina). Ecol. Indic. 11 (2), 240-247.

[g] Biljana, Š., Snežana, M., Milan, M., (2012). Multielement profiles of soil, road dust, tree bark and wood-rotten fungi collected at various distances from high-frequency road in urban area. Ecol. Indic. 13 (1), 168-177.

[h] Anugrah, R.W., Aaron, K.O., Kentaro, T., Ryuichi, S., Shigeru, O., (2012). Metal contents and Pb isotopes in road-side dust and sediment of Japan. J. Geochem. Explor. 118, 68-76.

[i] Jun, Y., Kumiko, Y., Ayumi, Y., Yuri, I., Takaya, K., Kodai, M., Mai, T., Atsushi, T., (2014). Lead and other elements in house dust of Japanese residences-source of lead and health risks due to metal exposure. Environ. Pollut. 189, 223-228.

Table S4 Continued

City	Ga	La	Mn	Nb	Ni	Pb	Rb	Sb	Sn	Sr	Th	Ti	U	V	Y	Zn	Zr
Xi'an, China [a]	14.54	31.92	486.42	11.90	21.04	93.45	44.19			186.54		2949.18		69.28	18.57	272.04	120.13
Xining, China[b]	18.54	19.71	377.69	12.95	23.47	62.82	58.41	8.91	22.81	245.08	11.16	1977.87	4.6	51.78	25.49	104.84	194.74
Beijing, China[c]					37.6	119								61.7		408	
Xi'an, China[d]			687			230.52		5.41								421.46	
Delhi, India[e]					36.4	120.7										284.5	
Buenos Aires, Argentina[f]			569		50	208										751	
Banja Luka, Serbia[g]			668		41.4	608										272	
Tokyo, Japan[h]					29.6	245										1888	
Tokyo, Japan[i]			226		59.6	57.9		10.1	18.4	66.8		0.15%Ti	0.40	24.7		920	

[a] This study.

[b] Li, XP, ZhangM, GaoY, ZhangYC, Zhang X, Yan XY, Wang S, Yang R, Liu B, Yu HT, (2018). Urban street dust bound 24 potentially toxic metal/metalloids (PTMs) from Xining valley-city, NW China: Spatial occurrences, sources and health risks, Ecotoxicol. Environ. Safe. 162, 474-487

[C] Tang, Y., Han, G.L., (2017). Characteristics of major elements and heavy metals in atmospheric dust in Beijing, China. J. Geochem. Explor. 176, 114-119.

[d] Yongming, H., Peixuan, D., Junji, C. and Posmentier, E.S., (2006). Multivariate analysis of heavy metal contamination in urban dusts of Xi'an, Central China. Sci. Total Environ., 355(1-3), 176-186.

[e] Suryawanshi, P.V., Rajaram, B.S., Bhanarkar, A.D., Chalapati, Rao, C.V., (2016). Determining heavy metal contamination of road dust in Delhi, India. Atmósfera 29 (3), 221-234.

[f] Fabián, G.F., Darío, R.G., Laura, D., Patricia, P., Ana, F., (2011). Metals associated with airborne particulate matter in road dust and tree bark collected in a megacity (Buenos Aires, Argentina). Ecol. Indic. 11 (2), 240-247.

[g] Biljana, Š., Snežana, M., Milan, M., (2012). Multielement profiles of soil, road dust, tree bark and wood-rotten fungi collected at various distances from high-frequency road in urban area. Ecol. Indic. 13 (1), 168-177.

[h] Anugrah, R.W., Aaron, K.O., Kentaro, T., Ryuichi, S., Shigeru, O., (2012). Metal contents and Pb isotopes in road-side dust and sediment of Japan. J. Geochem. Explor. 118, 68-76.

[i] Jun, Y., Kumiko, Y., Ayumi, Y., Yuri, I., Takaya, K., Kodai, M., Mai, T., Atsushi, T., (2014). Lead and other elements in house dust of Japanese residences-source of lead and health risks due to metal exposure. Environ. Pollut. 189, 223-228.

Table S5 The potential ecological risk assessment of street dust.

Index		As	Co	Cr	Cu	Mn	Ni	Pb	Sr	Ti	V	Zn
C_f^i	Max	3.27	1.89	10.46	14.46	0.94	3.83	12.57	1.57	1.02	3.48	62.07
	Min	0.22	0.75	1.73	0.73	0.62	0.21	1.77	0.33	0.58	2.22	1.47
	Mean	0.98	0.98	2.34	1.89	0.74	0.66	4.06	0.77	0.72	2.89	3.89
C_d	Max						77.65					
	Min						13.24					
	Mean						19.9					
E_r^i	Max	32.67	9.43	20.93	72.3	0.94	19.14	62.85	1.57	1.02	6.97	62.07
	Min	2.17	3.75	3.45	3.65	0.62	1.06	8.85	0.33	0.58	4.43	1.47
	Mean	9.78	4.9	4.67	9.43	0.74	3.29	20.32	0.77	0.72	5.77	3.89
RI	Max						135.29					
	Min						40.15					
	Mean						64.26					

Table S6 The correlation analysis of metals in Street dusts

	As	Ba	Ce	Co	Cr	Cu	Ga	La	Mn	Nb	Ni	Pb	Rb	Sr	Ti	V	Y	Zn	Zr
As	1.00																		
Ba	-0.11	1.00																	
Ce	-0.36**	0.63**	1.00																
Co	0.16	0.28**	0.27**	1.00															
Cr	0.14	0.27**	0.30**	0.52**	1.00														
Cu	0.27**	0.14	0.11	0.05	0.28**	1.00													
Ga	0.34**	0.15	0.12	0.13	0.31**	0.71**	1.00												
La	-0.02	0.24*	0.27**	0.05	0.20**	0.15	0.26**	1.00											
Mn	0.17	0.22*	0.16	0.43**	0.35**	0.12	0.10	0.02	1.00										
Nb	0.26**	0.28**	0.30**	0.27**	0.40**	0.52**	0.69**	0.19*	0.33**	1.00									
Ni	0.40**	0.15	0.19*	0.25**	0.45**	0.70**	0.90**	0.31**	0.29**	0.77**	1.00								
Pb	-0.11	0.44**	0.42**	0.41**	0.45**	0.51**	0.66**	0.26**	0.17	0.52**	0.59**	1.00							
Rb	0.29**	0.11	0.16	-0.18*	0.16	0.67**	0.84**	0.28**	0.01	0.62**	0.81**	0.45**	1.00						
Sr	0.24**	0.07	0.09	-0.12	0.01	0.63**	0.80**	0.16	0.10	0.46**	0.72**	0.36**	0.80**	1.00					
Ti	-0.04	0.44**	0.45**	0.17	0.35**	0.18	0.23**	0.35**	0.05	0.46**	0.26**	0.35**	0.29**	-0.03	1.00				
V	-0.14	0.42**	0.55**	0.23**	0.35**	0.06	0.18	0.42**	0.05	0.37**	0.22**	0.35**	0.23**	-0.12	0.80**	1.00			
Y	0.31**	0.21**	0.16	0.00	0.14	0.65**	0.85**	0.32**	0.04	0.62**	0.79**	0.53**	0.82**	0.81**	0.27**	0.15	1.00		
Zn	0.37**	0.14	-0.27**	0.18	0.05	0.29**	0.39**	0.01	0.12	0.21	0.32**	0.26**	0.10	0.23	-0.13	-0.23*	0.27**	1.00	
Zr	0.27**	0.18	0.19**	-0.12	0.20**	0.70**	0.86**	0.29**	-0.02	0.70**	0.81**	0.49**	0.93**	0.80**	0.35**	0.21*	0.89**	0.15	1.00

Notes: *Correlation is significant at the 0.05 level(2-tailed). **Correlation is significant at the 0.01 level(2-tailed).

Table S7 Varimax rotated factor matrix

Variable	Factor 1	Factor 3	Factor 4	Communalities
LogAs	0.32	0.25	**-0.66**	0.79
LogBa	0.11	0.21	**0.43**	0.78
LogCe	0.11	0.09	0.47	0.79
LogCo	-0.07	**0.81**	0.05	0.81
LogCr	0.17	**0.59**	-0.03	0.69
LogCu	**0.74**	0.15	0.05	0.62
LogGa	**0.93**	0.26	0.03	0.96
LogLa	0.24	0.05	0.07	0.41
LogMn	0.06	**0.49**	-0.12	0.62
LogNb	**0.66**	0.33	-0.11	0.85
LogNi	**0.87**	0.37	-0.14	0.96
LogPb	0.52	0.51	**0.58**	0.95
LogRb	**0.93**	-0.13	-0.06	0.96
LogSr	**0.90**	-0.11	0.05	0.96
LogTi	0.17	0.07	0.07	0.79
LogV	0.05	0.13	0.10	0.95
LogY	**0.91**	0.02	0.05	0.90
LogZn	0.28	**0.45**	-0.06	0.75
LogZr	**0.95**	-0.10	-0.01	0.98
% of Variance	34.80	11.71	6.69	
Cumulative %	34.80	61.14	67.83	

Extraction Method: Generalized Least Squares.
Rotation Method: Varimax with Kaiser Normalization, Rotation converged in 5 iterations.

Table S8 Daily exposure levels of children and adults to trace metals in dust under different exposure routes

	ADDing		ADDinh		ADDderm		LADD	
	Adults	Children	Adults	Children	Adults	Children	Adults	Children
As	2.09E-05	1.49E-04	3.08E-09	4.17E-09	6.18E-08	3.23E-07	1.05E-09	3.85E-10
Co	2.37E-05	1.69E-04	3.49E-09	4.73E-09	7.00E-08	3.66E-07	1.19E-09	4.06E-10
Cr	3.13E-04	2.24E-03	4.60E-08	6.25E-08	9.24E-07	4.83E-06	1.58E-08	5.36E-09
Cu	9.60E-05	6.86E-04	1.41E-08	1.92E-08	2.84E-07	1.48E-06		
Ni	3.86E-05	2.76E-04	5.67E-09	7.70E-09	1.14E-07	5.95E-07	1.95E-09	6.6E-10
Pb	1.69E-04	1.21E-03	2.48E-08	3.37E-08	4.99E-07	2.61E-06	8.51E-09	2.89E-09
Zn	4.11E-04	2.94E-03	6.05E-08	8.21E-08	1.21E-06	6.34E-06		
Sr	3.27E-04	2.34E-03	4.81E-08	6.53E-08	9.67E-07	5.05E-06		
Ba	1.30E-03	9.26E-03	1.91E-07	2.59E-07	3.83E-06	2.00E-05		
Mn	8.30E-04	5.93E-03	1.22E-07	1.66E-07	2.45E-006	1.28E-05		
V	1.18E-04	8.45E-04	1.74E-08	2.36E-08	3.49E-07	1.82E-06		
Total	3.65E-03	2.60E-04	5.36E-07	7.28E-07	1.08E-05	5.62E-05	2.85E-08	9.67E-09

Table S9 The risk index of children and adults of trace metals in dust under different exposure

Human	Element	HQing	HQinh	HQdermal	HI	Risk
Adults	As	6.97E-02	1.02E-05	5.02E-04	7.02E-02	1.59E-08
	Co	1.18E-03	6.10E-04	4.38E-06	1.79E-03	1.17E-08
	Cr	1.04E-01	1.61E-03	1.54E-02	1.21E-01	6.63E-07
	Cu	2.40E-03	3.51E-07	2.36E-05	2.42E-03	
	Ni	1.93E-03	2.75E-07	2.11E-04	2.14E-03	1.63E-09
	Pb	4.82E-02	7.06E-06	9.50E-04	4.92E-02	3.58E-10
	Zn	1.37E-03	2.02E-07	2.02E-05	1.39E-03	
	Sr	5.45E-04	8.02E-08	8.06E-06	5.53E-04	
	Ba	1.85E-02	1.33E-03	7.82E-04	2.06E-02	
	Mn	1.80E-02	8.54E-03	1.33E-03	2.79E-02	
	V	1.69E-02	2.48E-06	4.99E-03	2.19E-02	
	Total	2.83E-01	1.21E-02	2.42E-02	3.19E-01	6.92E-07
Children	As	4.98E-01	1.39E-05	2.62E-03	5.01E-01	5.40E-09
	Co	8.47E-03	8.29E-04	2.29E-05	9.32E-03	3.97E-09
	Cr	7.45E-01	2.18E-03	8.05E-02	8.28E-01	2.25E-07
	Cu	1.71E-02	4.77E-07	1.23E-04	1.72E-02	
	Ni	1.38E-02	3.74E-07	1.10E-03	1.49E-02	5.55E-10
	Pb	3.45E-01	7.40E-04	4.96E-03	3.51E-01	1.21E-10
	Zn	9.79E-03	2.74E-07	1.06E-04	9.90E-03	
	Sr	3.90E-03	1.09E-07	4.21E-05	3.94E-03	
	Ba	1.32E-01	1.81E-03	4.08E-03	1.38E-01	
	Mn	1.29E-01	1.16E-02	6.96E-03	1.48E-01	
	V	1.21E-01	3.37E-06	2.61E-02	1.47E-01	
	Total	2.02E+00	1.72E-02	1.27E-01	2.17E+00	2.35E-07
